# Case Report: Hereditary neuropathy with liability to pressure palsy (HNPP): the role of genetic investigation in diagnostic assessment

**DOI:** 10.3389/fgene.2025.1613022

**Published:** 2025-08-14

**Authors:** Salvatore Savasta, Fabiola Serra, Lucrezia Galimberti, Francesco Fabrizio Comisi, Marcello Cossu, Alessandro Vannelli, Maddalena Masala, Sara Tanca, Stefania Murru

**Affiliations:** ^1^ Pediatric and Rare Diseases Clinic, Microcitemico Hospital “A. Cao”, Department of Medical Sciences and Public Health, University of Cagliari, Cagliari, Italy; ^2^ Pediatric and Rare Diseases Clinic, Microcitemico Hospital “A. Cao”, University of Cagliari, Cagliari, Italy; ^3^ Binaghi Hospital, ASL8, Cagliari, Italy; ^4^ Genetic and Genomic Laboratory, Pediatric Children Hospital “A. Cao”, ASL 8, Cagliari, Italy

**Keywords:** HNPP, genetics, diagnosis, child, demyelinating, palsy, neuropathy, hereditary

## Abstract

Hereditary neuropathy with liability to pressure palsies (HNPP) is a genetic disorder characterized by recurrent focal neuropathies typically occurring at sites of nerve entrapment or compression. It is classically described as a painless condition; however, pain is frequently reported. Due to its rarity and variable clinical presentation, HNPP is often underdiagnosed or initially misdiagnosed. We report the case of a 14-year-old girl who presented with sudden-onset arm weakness and pain following physical activity. The clinical presentation initially raised suspicion for a hereditary demyelinating neuropathy. Although there was no known family history, the patient’s age and the persistence of symptoms supported the hypothesis of a genetic etiology. Neurophysiological studies were consistent with HNPP, which was subsequently confirmed by genetic testing. The primary aim of this report is to emphasize the importance of recognizing the early manifestations of HNPP—including pain, a symptom often underestimated or overlooked—in order to enable prompt diagnosis, reduce unnecessary diagnostic delays, and ensure timely initiation of appropriate genetic counseling. This case supports the notion that pain may represent an early feature of HNPP and should not lead clinicians away from considering this diagnosis.

## Introduction

Hereditary neuropathy with liability to pressure palsies (HNPP) is an autosomal dominant disorder characterized by recurrent peripheral mononeuropathies at sites of nerve entrapment, typically triggered by minor trauma or compression (Mouton et al., 1999). Although HNPP is classically described as a painless neuropathy (Mouton et al., 1999), recent studies have shown that pain is a frequent symptom. In particular, up to 75% of patients report experiencing pain during the disease course, and in approximately 12% of cases, pain represents the initial manifestation (Yilmaz et al., 2015).

First described in 1947 by Dutch neurologist De Jong, the condition was originally referred to as “potato grubbing disease,” in reference to the occupation of the first reported family, whose repetitive work led to compressive neuropathies (Beydoun and Cho, 2013; Attarian et al., 2020). The estimated prevalence of HNPP ranges from 7 to 16 per 100,000 individuals, though it seems to vary considerably across populations and studies (Beydoun and Cho, 2013; Attarian et al., 2020; van Paassen et al., 2014). In fact, more specific data report prevalence rates from approximately 0.84 per 100,000 in Ireland, 2 per 100,000 in Northern England, and 16 per 100,000 in Southwestern Finland. In contrast, a Korean newborn screening study revealed a markedly higher prevalence of 1 in 1,698 (Park et al., 2018): further studies on larger populations would be needed to know the true global prevalence. The clinical presentation typically involves recurrent sensory and motor mononeuropathies, most often beginning in the second or third decade of life (Attarian et al., 2020). Diagnosis is supported by nerve conduction studies, which reveal characteristic delays in distal motor latencies.

HNPP is caused by a deletion on chromosome 17p11.2–p12 involving the *PMP22* gene, which encodes peripheral myelin protein 22 (Verhagen et al., 1993). Reduced expression of *PMP22* leads to impaired myelin function and clinical manifestations of the disease (Beydoun and Cho, 2013). In addition to the common deletion, other rare mutations in the *PMP22* gene, such as point mutations and small deletions, have also been implicated in the pathogenesis of HNPP (Beydoun and Cho, 2013). The disorder is also referred to as “tomaculous neuropathy”, due to the presence of focal “sausage-like” myelin swellings known as tomacula (Saurabh and Ahmad, 2023).

Nerve biopsy, once commonly used for diagnosis, has largely been replaced by genetic testing (Beydoun and Cho, 2013).

The primary aim of this report is to highlight the importance of performing neurophysiological and genetic testing when clinical suspicion of HNPP arises, in order to achieve a definitive diagnosis. Particular attention should be given to the presence of pain, which—despite being frequently underrecognized—may represent an early and relevant symptom of the disease.

## Case report

A 14-year-old girl was initially admitted to another hospital due to the sudden onset of weakness and pain in her left arm upon awakening. She had played basketball in the preceding days. She was born at term following an uneventful pregnancy and spontaneous delivery. At 4 months of age, she had been hospitalized for bronchiolitis. No other significant medical history was reported, and her psychomotor development had been normal. There was no family history of neurological disorders, and her parents were not consanguineous.

Neurological examination revealed proximal weakness of the left upper limb, with compensatory recruitment of the parabrachial muscles during extension, flexion, and abduction. Localized tenderness was noted on palpation near the distal head of the biceps tendon, while distal muscle strength remained preserved. Initial blood tests (including complete blood count, creatine phosphokinase, electrolytes, and C-reactive protein) were within normal limits. Cervical spine and left shoulder X-rays were unremarkable. Additional investigations including orthopedic evaluation, brain CT scan, and ophthalmologic examination also yielded normal results. Brain and cervical-thoracic spine MRI revealed a mild dilation of the ependymal canal in the thoracic region between T3–T4 and T8–T9.

An electromyography (EMG) study showed increased distal motor latency of the median and peroneal nerves bilaterally, reduced motor conduction velocities in the median, ulnar, peroneal, and tibial nerves bilaterally, and decreased sensory conduction velocities in the examined nerves. These findings were suggestive of a hereditary demyelinating polyneuropathy of probable genetic origin.

**FIGURE 1:**
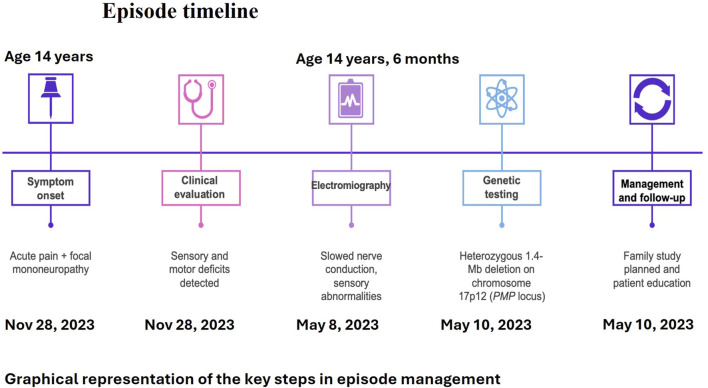
Episode timeline.

Five months later, the patient was referred to our center for further diagnostic evaluation, as her symptoms had improved but not completely resolved. On admission, her neurological examination was normal; although she reported lingering weakness in her left arm, both muscle strength and sensory function were preserved. Repeat blood tests were within normal limits. Shoulder MRI and ultrasound showed no abnormalities. Neurophysiological assessment revealed normal somatosensory evoked potentials. However, EMG and nerve conduction studies demonstrated a chronic, widespread, mixed sensorimotor neurogenic impairment with demyelinating features. The left ulnar nerve at the elbow showed reduced sensory conduction velocity (38.4 m/s) and motor conduction velocity ranging from 36.4 to 27.4 m/s. Similarly, the right common peroneal nerve showed reduced motor conduction velocities at the fibular head (39.7 m/s) and across the knee (29.9 m/s). These findings were consistent with hereditary neuropathy with liability to pressure palsies (HNPP). Genetic testing subsequently confirmed a heterozygous 1.4 Mb deletion on chromosome 17p12, encompassing the *PMP22* gene. Upon discharge, genetic counseling was provided, along with behavioral recommendations aimed at reducing the risk of symptom recurrence (Figure 1).

## Methods

**FIGURE 2:**
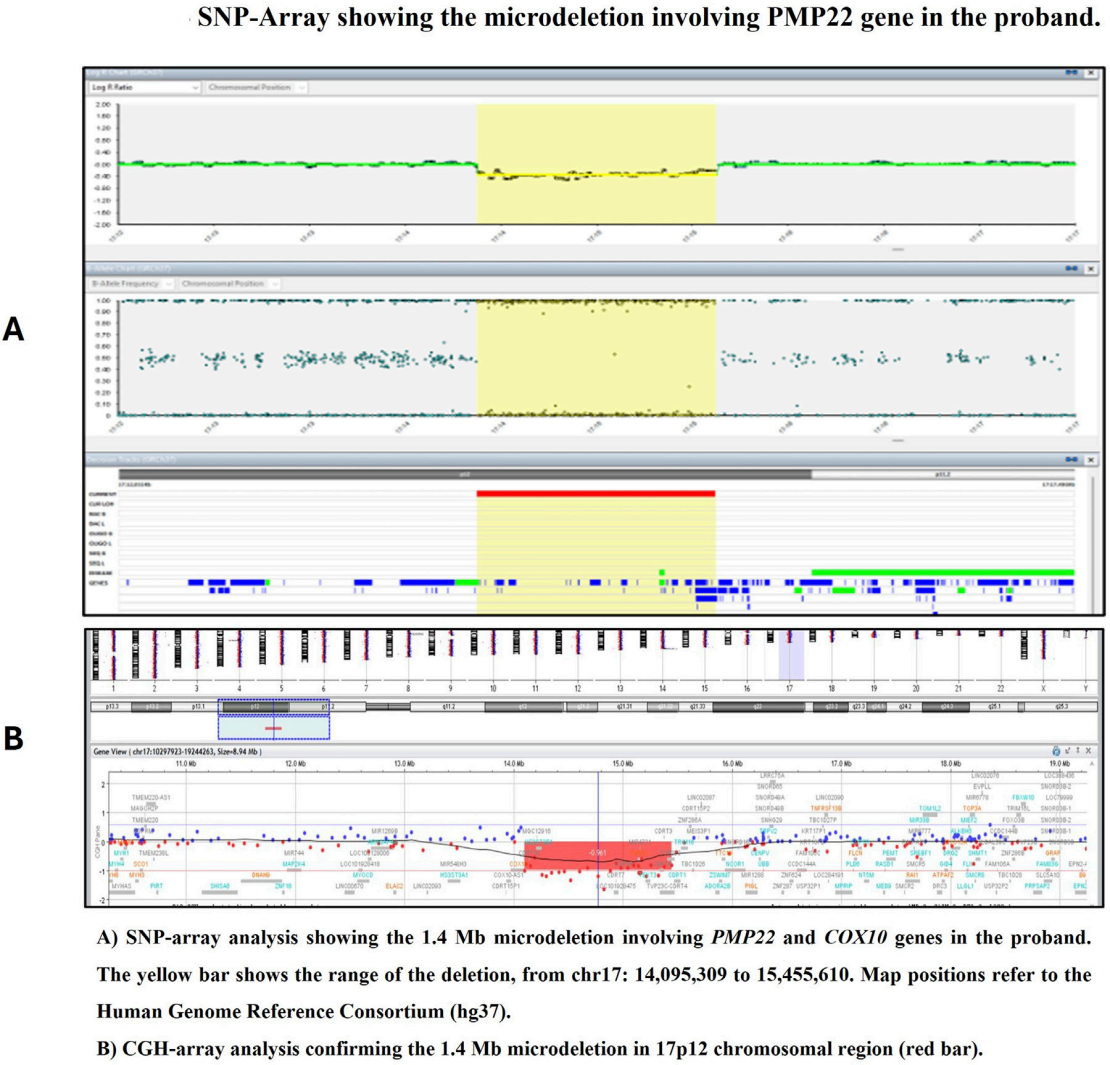
SNP-Array showing the microdeletion involving PMP22 gene in the proband. **(A)** SNP-array analysis showing the 1.4 Mb microdeletion involving PMP22 and COX10 genes in the proband. The yellow bar shows the range of the deletion, from chr17: 14,095,309 to 15,455,610. Map positions refer to the Human Genome Reference Consortium (hg37). **(B)** CGH-array analysis confirming the 1.4 Mb microdeletion in 17p12 chromosomal region (red bar).

A written informed consent for blood sample collection and genetic analysis was obtained from the proband in compliance with the Declaration of Helsinki. Genomic DNA was extracted from peripheral blood samples using the QIAamp DNA Blood Kit (Qiagen, Hilden, Germany) following the manufacturer’s protocol. SNP-Array was performed using the Infinium CytoSNP 850K v1.4 microarray, spanning the entire genome, with a resolution of 100 kb, according to the instruction provided by the manufacturer. The Illumina Infinium CytoSNP 850K v1.4 Bead Chip platform and scanning using NextSeq550 was used for SNP-Array analysis. The level of resolution, dependent on the number of SNPs and their distance in the investigated DNA region is between 5 and 1 Kb in areas including relevant genes as indicated by ICCG (International Collaboration for Clinical Genomics) and CCMC (Cancer Cytogenomics Microarray Consortium). The analysis of CNVs (Copy Number Variants) and regions with LOH (loss of heterozygos-ity) is performed using Bluefuse Multi Software v4.3. Map positions refer to the Human Genome Reference Consortium (hg19). Genetic testing identified a heterozygous interstitial microdeletion encompassing 1.4 Mb in the short arm of chromosome 17 with breakpoints in band 17p12 (arr [GRCh37] 17p12 (14095309_15455610)). This region contains the *PMP22* gene (Figure 2). Microdeletion was confirmed by Comparative Genomic Hybridization (CGH) Array method using Agilent GenetiSure Cyto CGH + SNP 4 × 180k.

## Discussion

HNPP is an autosomal dominant disorder that is often underdiagnosed (Dubourg et al., 2000). Some studies have highlighted a higher prevalence in males (male-to-female ratio of 1.3:1), probably due to greater exposure to trauma in this population (Tinant et al., 2002). Approximately one-third of individuals carrying the deletion remain asymptomatic, while most symptomatic cases follow a favorable course (Tinant et al., 2002). A family history of compressive neuropathies may be absent in HNPP patients, either due to sporadic mutations or the presence of asymptomatic carriers (Beydoun et al., 2008). The classic presentation of HNPP is characterized by recurrent, transient focal mononeuropathies (Pareyson et al., 1996).

Symptoms are typically triggered by prolonged compression, chronic mild pressure, or trauma (Felice et al., 1999). The age of onset varies widely, ranging from infancy to adulthood, with most cases occurring between 10 and 30 years of age (Mouton et al., 1999; Beydoun and Cho, 2013). In 85% of cases, the initial clinical manifestation is acute and painless; however, as the disease progresses, up to 75% of patients develop persistent pain (Attarian et al., 2020; Beales et al., 2017). According to the literature, our patient falls within the typical age range and experienced a stress-related event preceding symptom onset. However, the presence of pain at onset is uncommon in patients diagnosed before 18 years of age, as highlighted in a recent series of seven pediatric cases (Isik and Odabaşı, 2024). In patients presenting with pain at disease onset, the clinical phenotype may differ from the classical painless presentation, and for this reason diagnosis is often delayed. Symptoms may present with neuropathic or musculoskeletal features, leading to consideration of alternative diagnoses, such as fibromyalgia, when features such as allodynia and widespread discomfort overlap (Yilmaz et al., 2015; Cotelli et al., 2023). Although standard nerve conduction studies remain the principal method for diagnosis, no specific electrophysiological pattern has yet been identified for this presentation. In some cases, initial studies may show only subtle sensory abnormalities or even normal findings, potentially delaying diagnosis (Yilmaz et al., 2015; Cotelli et al., 2023). The nerves most frequently affected by compressive trauma include the ulnar nerve (at the elbow), the peroneal nerve (at the fibular head), and the radial nerve (at the spiral groove), followed by the brachial plexus and median nerve (Potulska-Chromik et al., 2014). Cranial nerve involvement is rare (van Paassen et al., 2014; Dubourg et al., 2000). In approximately 85% of patients, a heterozygous 1.5-Mb deletion on chromosome 17p12 (*PMP22* locus) is identified (Beydoun and Cho, 2013). The same locus is duplicated in Charcot–Marie–Tooth disease type 1A, which is also the main differential diagnosis (Yilmaz et al., 2015). The PMP22 protein is involved in myelin production by Schwann cells and plays an essential structural role in stabilizing the myelin sheath, thereby protecting nerves from repetitive trauma (Beydoun and Cho, 2013; Attarian et al., 2020; Li et al., 2013a). Loss of function can result from point or frameshift mutations in about 15% of cases, while *de novo* mutations account for approximately 20% (Beydoun and Cho, 2013; Saurabh and Ahmad, 2023; Pareyson et al., 1996; Li et al., 2013a). In our case, genetic testing confirmed the diagnosis with a heterozygous 1.4-Mb deletion on chromosome 17p12. A negative family history, as seen in our patient, is common, since approximately half of deletion carriers remain asymptomatic (van Paassen et al., 2014; Infante et al., 2001). Further investigations, including a segregation study and electromyography of the patient’s parents, are planned to determine whether the deletion is inherited or *de novo*.

While mechanical compression and trauma are recognized as the main triggers for symptom onset in HNPP, several studies have suggested additional modifying factors that may influence disease penetrance. Metabolic stressors, such as diabetes mellitus, hypothyroidism, or vitamin deficiencies, have been proposed as potential risk factors that may unmask subclinical neuropathy in genetically predisposed individuals (Sung et al., 2012; Luigetti et al., 2014). Furthermore, environmental and occupational exposures (for example, repetitive movements, poor ergonomics, or cold temperatures) may contribute to increased vulnerability (Kim et al., 2010). In our patient we detected insufficient Vitamin D levels (11.3 ng/mL) as a side ward, but further studies would be needed to understand whether this could influence symptom onset.

Conversely, potential protective factors associated with the asymptomatic phenotype have been hypothesized but are less well-defined. One proposed factor is the presence of compensatory mechanisms in peripheral myelination, including upregulation of other myelin-related genes or structural variants in modifier loci, though evidence remains preliminary (Li et al., 2013b). Additionally, lifestyle factors that reduce nerve compression risk, such as regular physical activity without overuse, may contribute to the absence of clinical symptoms in some carriers (Kimura, 2013). A better understanding of these modifiers could help stratify risk and guide preventive strategies.

Neurophysiological studies typically show prolonged distal motor latencies, especially in the median and peroneal nerves (van Paassen et al., 2014; Li et al., 2002). Focal motor slowing at entrapment sites is common, particularly in the ulnar nerve at the elbow, though it may also affect the peroneal nerve at the fibular head (van Paassen et al., 2014; Li et al., 2002). Distal sensory conduction velocities are often diffusely reduced, findings that can be present in both symptomatic and asymptomatic nerves (Mouton et al., 1999; Beydoun and Cho, 2013; van Paassen et al., 2014; Li et al., 2002). Acute neuropathies usually resolve within days to weeks (Dubourg et al., 2000). Full recovery occurs in approximately 50% of episodes; if chronic symptoms persist, they are usually mild (van Paassen et al., 2014). However, relapses can be frequent, and paresis may last several months, as observed in our patient (Beydoun and Cho, 2013; Attarian et al., 2020; Dubourg et al., 2000). When symptoms persist or recur, patients may develop distal atrophy, sometimes symmetrical, resembling a CMT phenotype (Attarian et al., 2020; van Paassen et al., 2014; Chance, 2006). Life expectancy remains unaffected (van Paassen et al., 2014). To date, no pharmacological treatment has proven effective, and management remains symptomatic (Attarian et al., 2020; Beales et al., 2017; Potulska-Chromik et al., 2014). Patient education is crucial to prevent recurrences by avoiding positions that promote nerve compression, such as leg-crossing, repetitive wrist movements, prolonged leaning on the elbows, or rapid weight loss (Attarian et al., 2020; van Paassen et al., 2014; Potulska-Chromik et al., 2014). Upon discharge, we provided the patient and her family with the information they needed to understand the origin and course of the disease, reassuring them that it was benign and that she could lead a completely normal life. We also provided her with advice to prevent further episodes, and we prescribed a neurotrophic agent.

## Conclusion

Hereditary neuropathy with liability to pressure palsies (HNPP) is a well-known condition that remains significantly underdiagnosed. Neurophysiological studies can reveal sensorimotor demyelinating lesions in affected nerves, while histological examination may demonstrate characteristic myelin thickening (tomacula), resulting from hypermyelination due to PMP22 protein deficiency*.* Genetic testing now provides a reliable, non-invasive method for confirming the diagnosis, largely replacing the need for nerve biopsy. As this is a single case report, our primary objective was to describe and contextualize a rare clinical presentation within the existing literature, rather than to establish generalizable findings. The case presented highlights not only the frequent delays in HNPP diagnosis, but also the undervalued role of pain as an early symptom. In our patient, several months elapsed before the correct diagnosis was established. Since diagnosis can be challenging, pain should be recognized as a possible presenting symptom, as it can lead to an early genetic diagnosis and help avoid unnecessary investigations.

Given its generally favorable prognosis, early identification of HNPP enables patients and their families to manage the condition effectively and maintain a normal quality of life.

## Data Availability

The original contributions presented in the study are publicly available. This data can be found here: (https://www.ncbi.nlm.nih.gov/clinvar/submitters/510099).
